# Loss of LMO4 in the Retina Leads to Reduction of GABAergic Amacrine Cells and Functional Deficits

**DOI:** 10.1371/journal.pone.0013232

**Published:** 2010-10-07

**Authors:** Philippe M. Duquette, Xun Zhou, Nida Lerma Yap, Erik J. MacLaren, Jesse J. Lu, Valerie A. Wallace, Hsiao-Huei Chen

**Affiliations:** 1 Centre for Stroke Recovery, Neuroscience, Ottawa Hospital Research Institute, Ottawa, Ontario, Canada; 2 Molecular Medicine Program, Ottawa Hospital Research Institute, Ottawa, Ontario, Canada; 3 Department of Medicine, University of Ottawa, Ottawa, Ontario, Canada; 4 Department of Cellular and Molecular Medicine, University of Ottawa, Ottawa, Ontario, Canada; 5 Department of Biochemistry, Microbiology and Immunology, University of Ottawa, Ottawa, Ontario, Canada; 6 University of Ottawa Eye Institute, University of Ottawa, Ottawa, Ontario, Canada; University of Dayton, United States of America

## Abstract

**Background:**

LMO4 is a transcription cofactor expressed during retinal development and in amacrine neurons at birth. A previous study in zebrafish reported that morpholino RNA ablation of one of two related genes, LMO4b, increases the size of eyes in embryos. However, the significance of LMO4 in mammalian eye development and function remained unknown since LMO4 null mice die prior to birth.

**Methodology/Principal Findings:**

We observed the presence of a smaller eye and/or coloboma in ∼40% LMO4 null mouse embryos. To investigate the postnatal role of LMO4 in retinal development and function, LMO4 was conditionally ablated in retinal progenitor cells using the Pax6 alpha-enhancer Cre/LMO4flox mice. We found that these mice have fewer Bhlhb5-positive GABAergic amacrine and OFF-cone bipolar cells. The deficit appears to affect the postnatal wave of Bhlhb5+ neurons, suggesting a temporal requirement for LMO4 in retinal neuron development. In contrast, cholinergic and dopaminergic amacrine, rod bipolar and photoreceptor cell numbers were not affected. The selective reduction in these interneurons was accompanied by a functional deficit revealed by electroretinography, with reduced amplitude of b-waves, indicating deficits in the inner nuclear layer of the retina.

**Conclusions/Significance:**

Inhibitory GABAergic interneurons play a critical function in controlling retinal image processing, and are important for neural networks in the central nervous system. Our finding of an essential postnatal function of LMO4 in the differentiation of Bhlhb5-expressing inhibitory interneurons in the retina may be a general mechanism whereby LMO4 controls the production of inhibitory interneurons in the nervous system.

## Introduction

The vertebrate retina derives from common, multipotent progenitor cells that give rise to six classes of neurons and one type of glial cell organized into three nuclear layers. The rod and cone photoreceptors reside in the outer nuclear layer (ONL), three types of interneurons including the horizontal, bipolar and amacrine neurons, together with the Müller glial cells are found in the inner nuclear layer (INL) and the displaced amacrine and ganglion cells are in the ganglion cell layer (GCL) [1], [2]. The photoreceptors are the primary sensory neurons responding to light stimuli, the interneurons process a highly complex array of spatial and frequency information and convey this information to the retinal ganglion cells, the final output neurons of the retina. Mammalian retinal development starts at mid-gestation (embryonic day 12 for the mouse) and continues into the first two weeks after birth. Retinal ganglion cells, amacrine cells, cone photoreceptors, and horizontal cells differentiate first and the process is almost complete at birth (gestation period being 21 days in mice). In contrast, bipolar cells, rod cells and Müller glial cells continue to be generated from neural precursors up to two weeks after birth (see review [2]).

Many transcription factors specify retinal cell fate, and their precise hierarchy is gradually yielding to genetic analysis (for review, see [2]). Homeodomain factors are thought to regulate the layer specificity but not the neuronal fate, while basic helix loop helix (bHLH) transcription factors determine the neuronal fate within homeodomain factor-specified layers [2]–[4]. Thus, combinations of proper bHLH and homeodomain factors are required for neuronal subtype specification.

Amacrine cells are the most complex component of the vertebrate retina, comprising almost 40 different functional types based on their neurotransmitters and their synaptic partners. The bHLH genes Math3 and NeuroD are expressed in retinal progenitors at embryonic stages when amacrine cells are generated [2], [5], [6]. Even though Math3/NeuroD double mutant embryos exhibit selective loss of amacrine cells, misexpression of either Math3 or NeuroD only promotes the generation of rod photoreceptor cells [5]. However, misexpression of Math3 or NeuroD together with the paired box homeodomain protein Pax6 promotes amacrine cell genesis [5], suggesting that the combination of bHLH and the homeobox genes is important for amacrine cell fate determination. For example, Bhlhb5 is required in the specification of GABAergic amacrine cells [7]. The transcriptional mechanisms that control Bhlhb5 expression are not well understood, but likely involve NeuroD/Math3. On the other hand, ablation of Pax6 in retinal neural progenitors leads to the production of only amacrine cells [8], indicating that amacrine cell differentiation is complex and is likely to require additional factors.

Rod and cone bipolar cells transmit signals from rod and cone photoreceptor to retinal ganglion cells, respectively. There are one rod bipolar and nine cone bipolar cell types. Bipolar cell fate specification is also dependent upon the combined action of the homeodomain factor Chx10 and the bHLH factors Mash1 and Math3 [4], [9]. Recent studies further showed that Bhlhb4 is required for production of rod bipolar cells [10] whereas Bhlhb5 is required for type 2 Off-cone bipolar cells [7].

The LIM domain only 4 protein is a transcription cofactor that interacts with many transcription factors and modulates their activity [11]–[21]. LMO4 augments the transcriptional inhibitory activity of the bHLH factor HEN1 in cultured hippocampal neurons and controls neurite outgrowth [19]. LMO4 is essential for development of the central nervous system [22], [23]. Mice deficient in LMO4 die prior to birth and 50% of embryos display exencephaly [24]–[26]. Ablation of LMO4 in cortical neurons impairs thalamocortical projections to layer 5 [13]. In the spinal cord, LMO4 specifies the selection of interneuron excitatory and inhibitory cell fate [20]. Ablation of one of the two paralogs of LMO4 in zebrafish increases the size of the eye [27]. In the chick, LMO4 augments Wnt signaling in the progenitors of the ciliary marginal zone of the retina [28]. LMO4 is expressed in the amacrine cell layer of the postnatal day 0 mouse retina [29], but the functional importance of LMO4 in the development of the mammalian retina has not been studied.

In characterizing the phenotype of LMO4 deficient mice we noticed a structural defect in the eye. This defect, coloboma, results from incomplete closure of the optic fissure during development [30]. To understand the functional significance of LMO4 in mouse retinal development, we conditionally ablated a floxed LMO4 allele in the retina using the Pax6 alpha enhancer driven Cre recombinase [8]. Whereas these mice do not develop coloboma and have normal-sized eyes, using neuron subtype-specific markers, we observed a reduction in GABAergic amacrine and OFF-cone bipolar cells. Electroretinograms revealed a functional deficit in these mice.

## Materials and Methods

### Animals

All experiments in animals were approved by the University of Ottawa animal care ethics committee, adhering to the guidelines of the Canadian Council on Animal Care under the protocol permit OGH/RI-36. The Pax6 α-Cre transgenic mice with a downstream GFP reporter tag were obtained from P. Gruss [8] and were maintained on a C57BL/6 background. α-Cre/wt mice were crossed to homozygote LMO4flox/flox mice [24] to generate F1 heterozygotes that were backcrossed to produce α-Cre/LMO4flox homozygote mutant or littermate control (LMO4flox/flox) mice. LMO4 null mice were described previously [26]. Genotyping for the α-Cre transgene, LMO4flox or LMO4 null alleles was performed by PCR.

### Immunohistochemistry

Embryos and dissected eyes plus optic nerves from animals younger than P7 were fixed in 4% paraformaldehyde (PFA) overnight in 0.1 M phosphate buffered saline (PBS) pH 7.4. Older animals were transcardially perfused with 4% PFA prior to overnight incubation. Following PBS washes, samples were cryoprotected overnight in a 30% sucrose/PBS solution. Tissues were embedded in a 1∶1 mix of 30% sucrose and OCT (Tissue-Tek, Japan) and frozen by immersion in −30°C 2-methyl butane. Embedded tissue was sectioned on a cryostat at 14 µm. Sections were transferred onto Superfrost Plus coated slides (Fisher Scientific, USA) and stored at −80°C. IHC was performed in sections at the level of the optic nerve plane and immunostaining was compared at the peripheral retinal (approximately up 500 µm from the tip) according to the protocol for fluorescence detection described previously (43,44). The following primary antibodies were used: goat polyclonal anti-Bhlhb5 (1/100), anti-LMO4 (c15) (1/50), anti-Brn3b (c13) (1/100, Santa Cruz Biotechnology), anti-choline acetyltransferase (ChAT) (1/2000, Millipore); sheep polyclonal anti-Chx10 (1/2000, Exalpha Biologicals, Inc); rabbit polyclonals anti-calretinin (1/2000, Swant), anti-tyrosine-hydroxylase (TH) (1/2000, ImmunoStar), anti-Prox1 (1/3000, Chemicon International), anti-phosphorylated histone 3 (Ser-10) (1/400, Upstate) and anti-activated caspase 3 (Asp175) (1/100, Cell Signaling; kind gift from David Park); mouse monoclonals anti-Pax6 (1/50; Developmental Studies Hybridoma Bank), anti-PKC (1/100, BD Pharmingen), anti-calbindin (1/500, Sigma), anti-rhodopsin (1/3; Developmental Studies Hybridoma Bank), anti-peanut agglutinin (PNA) (1/500, Santa Cruz Biotechnology), anti-HPC (syntaxin)(1/2000, Sigma). Antibodies were visualized with appropriate secondary antibodies conjugated with Cy3 and Cy2 and visualized on a Zeiss Z1 fluorescence microscope. For LMO4, Pax6, Brn3b and PKC immunostaining, antigen was unmasked with microwave treatment in 10 mM citrate buffer (in 1L water add 42g citric acid, 21 g Sodium hydroxide, pH = 6) using a 1000 watt microwave set at power 6 for 10 min and sections were cooled on ice for 10 min before processing for immunostaining.

To compare gene expression and differentiation, immunostained positive cells in the same region of the “peripheral” retina at the levels of the optic nerve plane were counted using Zeiss AxioVision automatic software. For every section, all immunopositive cells of a square of fixed area within 500 µm from the tip of retina were counted. For PKC+ rod bipolar and Bhlhb5+ amacrine and cone bipolar cells, cells were counted manually. At least 3 comparable retinal sections from three eyes from mutant and littermate wild type mice were used for statistical analysis by Student t test and p<0.05 were considered to be significant.

### *In situ* hybridization

Tissues were processed for in situ hybridization with digoxigenin-labeled antisense or sense riboprobes, as previously described [31]. For dual in situ hybridization and immunohistochemistry, biotin-labeled LMO4 antisense riboprobes were used, followed by biotin/avidin ABC kit (Vector laboratory) and cy2- conjugated streptavidin (Jackson laboratory) together with anti-Bhlhb5 goat antibody and cy3-conjugated anti-goat secondary antibody (Jackson laboratory). For more reliable comparisons of gene expression patterns, wild-type and mutant tissues were processed on the same slides.

### Electroretinography (ERG)

ERG was performed using the ESPION system (Diagnosys LLC, Littleton, MA) as previously described [32], [33]. Briefly, mice that had been dark-adapted overnight were anaesthetized under safe light conditions using an intraperitoneal injection of avertin at 250 mg/kg. Eyes were dilated using both 1% tropicamide and 2.5% phenylephrine hydrochloride (Alcon Canada). To ensure constant body temperature during testing, mice were put on a warming source. Silver wire loop electrodes were placed on both corneas with a drop of 0.3% hypromellose (Novartis) to maintain corneal hydration. A gold minidisc reference electrode was placed on the tongue and a ground needle electrode was placed subcutaneously in the tail. The animal's head was positioned under the center of the Ganzfeld dome. Single flash stimuli (4 ms duration) were presented at 11 increasing intensities ranging from 0.001 to 25 cd s/m2. Five ERG traces were obtained and averaged for each luminance step. The minimum negativity occurring between 10 and 40 ms post-stimulus was defined as the a-wave. The maximum positivity occurring between 40 and 80 ms poststimulus was defined as the b-wave [33]. Differences during ERG analyses were determined using ANOVA statistical analysis.

### Bhlhb5 promoter activity assay

Using a mouse targeting vector Bhlhb5-lacZ [7] (gift of Dr. Lin Gan, University of Rochester, NY, USA), a 2,110 bp fragment containing sequence from −1,898 to +212 relative to the start site of transcription was amplified by PCR using sequence-specific oligonucleotides Bhlhb5-fwd 5′-TTCTCGAGGGAGCCTCTCATTAGC-3′ and Bhlhb5-rev 5′-ATAAGCTTCCAGCCGACGGTGCT-3′. The PCR product was cloned between the XhoI and HindIII sites of the pGL3-basic vector and transfected into F11 neuronal cells using Lipofectamine 2000 as described previously [12] with control shRNA or shRNA specific to LMO4. LMO4shRNA (clone ID TRCN0000084375) and control scrambled non-silencing shRNA were purchased from OpenBiosystems. Transfection efficiency was normalized using a CMV enhancer-driven beta-galactosidase reporter plasmid as described [11].

## Results

### LMO4 null mice have developmental eye defects

**Figure 1:**
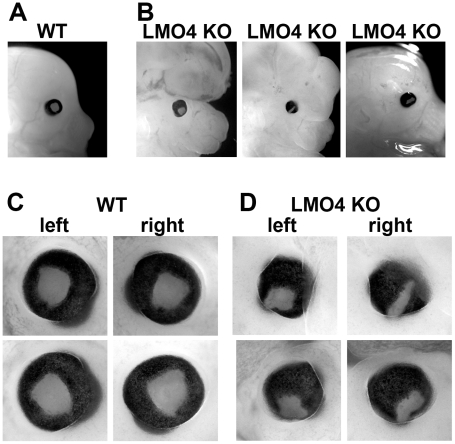
LMO4 null mice have a partially penetrant neural tube closure defect and coloboma. (A) Wild type mouse at embryonic day 14.5 (E14.5) shows normal forebrain and eye development. (B) Three phenotypically divergent LMO4 null (LMO4 KO) mouse embryos reveal exencephaly with relatively normal eye (left panel), exencephaly with a small eye and coloboma (middle panel), and non-exencephalic phenotype but with coloboma. (C & D) Close up views of the left and right eyes of wild type and LMO4 KO mice, respectively.

In phenotyping LMO4 null mice, we identified several features of altered eye development including smaller eyes and the presence of a coloboma phenotype in ∼40% of embryos (Figure 1). Although exencephaly was often associated with the eye phenotype, defective eye development was noted in some LMO4 null mice without exencephaly (Figure 1B, rightmost panel), indicating that these phenotypes are independent or only partially penetrant.

### LMO4 is expressed in the retina during development

**Figure 2:**
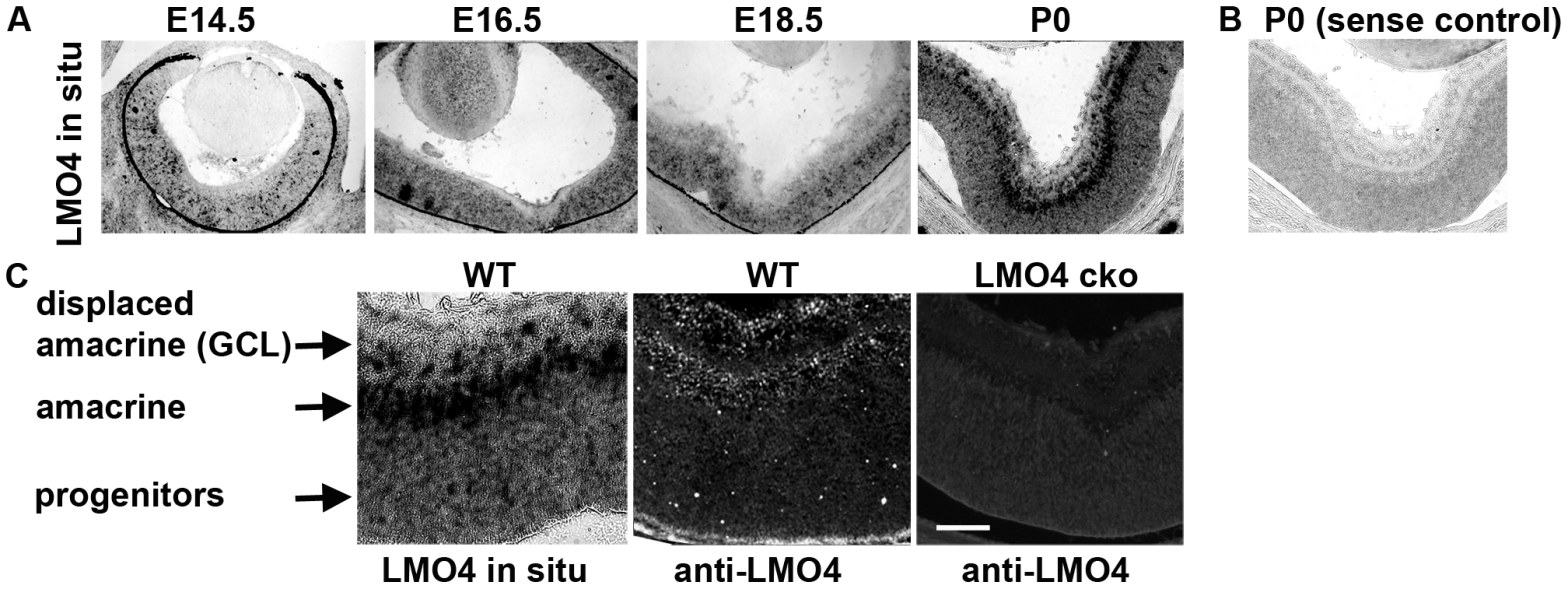
Pattern of LMO4 expression during retinal development. (A) In situ hybridization reveals gradually elevated expression of LMO4 mRNA from E14.5 to postnatal day 0 (P0). (B) Sense control probe shows background staining to be compared to the antisense probe used in (A). (C) Close-up view of P0 wild type (WT) retina shows high LMO4 mRNA levels in the displaced amacrine and amacrine layers and lower diffuse expression in the retinal progenitors. Immunofluorescence with anti-LMO4 antibody shows associated pattern of LMO4 expression in a P0 wt retina section and background staining in the LMO4 α-Cre conditionally ablated retina (LMO4 cko). Scale bar, 100 µm.

**Figure 3:**
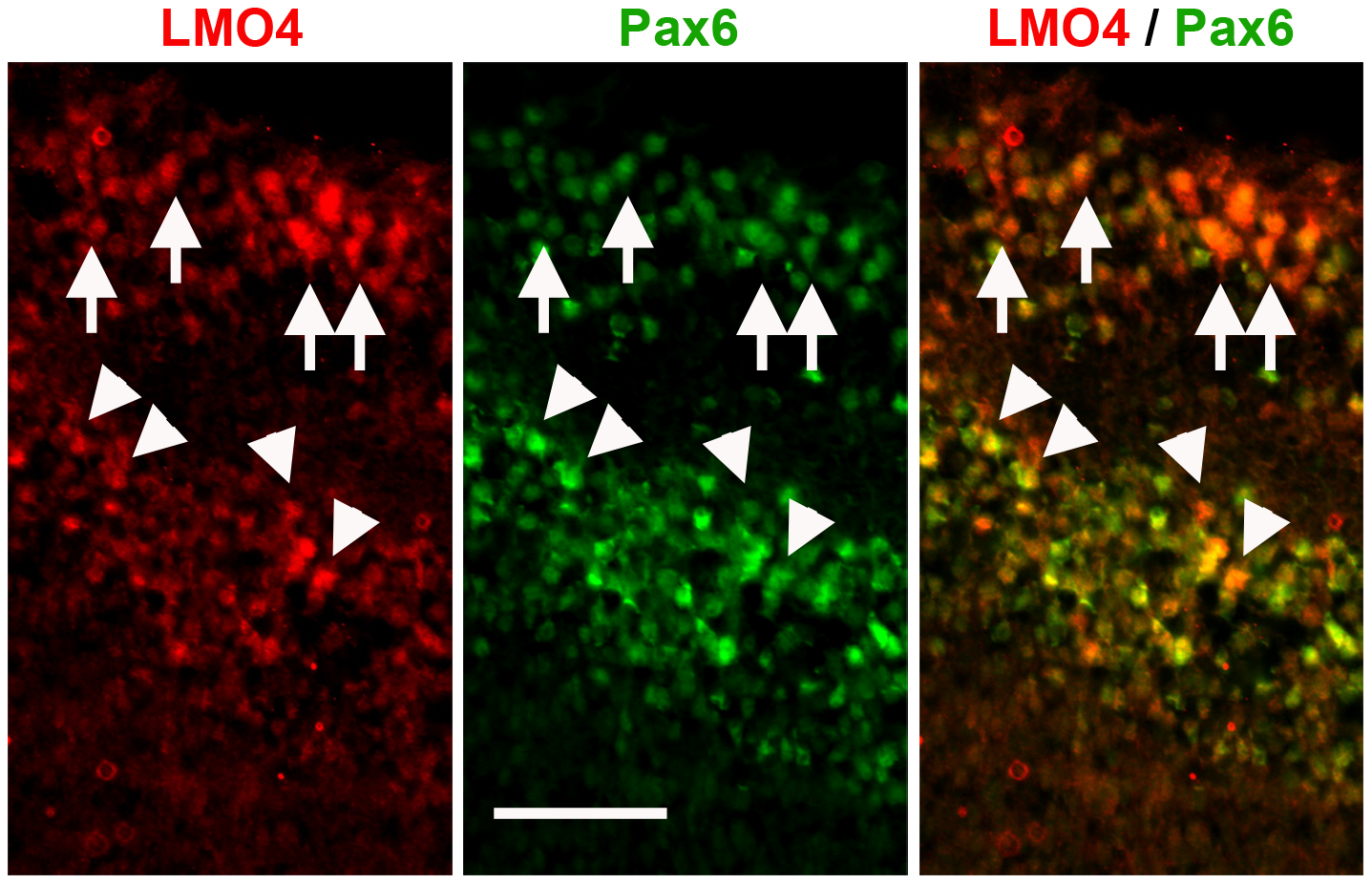
LMO4 is colocalized with Pax6 in amacrine and displaced amacrine cells. Dual immunofluorescence staining reveals LMO4 cells in red, Pax6 cells in green and dual labeled cells appear yellow. Amacrine cells are indicated by arrowheads and displaced amacrine cells in the retinal ganglion cell layer by arrows. Scale bar, 50 µm.

In situ hybridization analysis revealed that LMO4 mRNA is expressed in retinal progenitor cells at embryonic day 14.5 (E14.5) and its expression increases as the retina develops (Figure 2A). At E14.5, we were unable to detect LMO4 protein with the LMO4 antibody. However, at postnatal day 0 (P0), LMO4 mRNA and protein were found to be highly expressed in the amacrine cells at the inner nuclear layer, in displaced amacrine cells at the retinal ganglion cell layer and lower levels were detected in retinal progenitor cells (Figure 2C). Immunostaining at P0 showed that LMO4 is co-expressed with the pan-amacrine cell marker Pax6 at the inner nuclear layer (arrowheads, Figure 3) and in displaced amacrine cells at the retinal ganglion layer (arrows, Figure 3). LMO4 protein expression was again below detectable levels at P15 when the retinal layers and neuronal subtypes are fully developed (data not shown).

### Reduced Bhlhb5+ amacrine and bipolar cells in the peripheral retina of alpha-Cre/LMO4flox mice

Retinal cell differentiation begins at around E12 in mouse and continues into the first 2 postnatal weeks [2]. Because LMO4 null mouse embryos die before birth, it was not possible to study the requirement for this gene in postnatal retina function in this mouse strain. Thus, we conditionally ablated LMO4 in the developing retina by crossing LMO4flox mice with α-Cre-IRES-GFP transgenic mice where the Cre recombinase and a GFP reporter are driven by the retinal neural progenitor-specific Pax6 enhancer (Pax 6 alpha enhancer) [34]. α-Cre transgene is active in the peripheral retinal progenitors of the embryo and stably expressed in mature amacrine cells [8]. We confirmed expression of the GFP reporter predominantly in the ventral peripheral retinal progenitor cells at E14.5 (data not shown). By P0, all amacrine cells expressed the GFP reporter (and by inference the Cre-recombinase) (Figure S1). Immunostaining showed that LMO4 is ablated in the peripheral retina of the α-Cre/LMO4flox mice at P0; to simplify, we abbreviate these as LMO4 conditional knockout (LMO4 cko) mice (Figure 2C).

**Figure 4:**
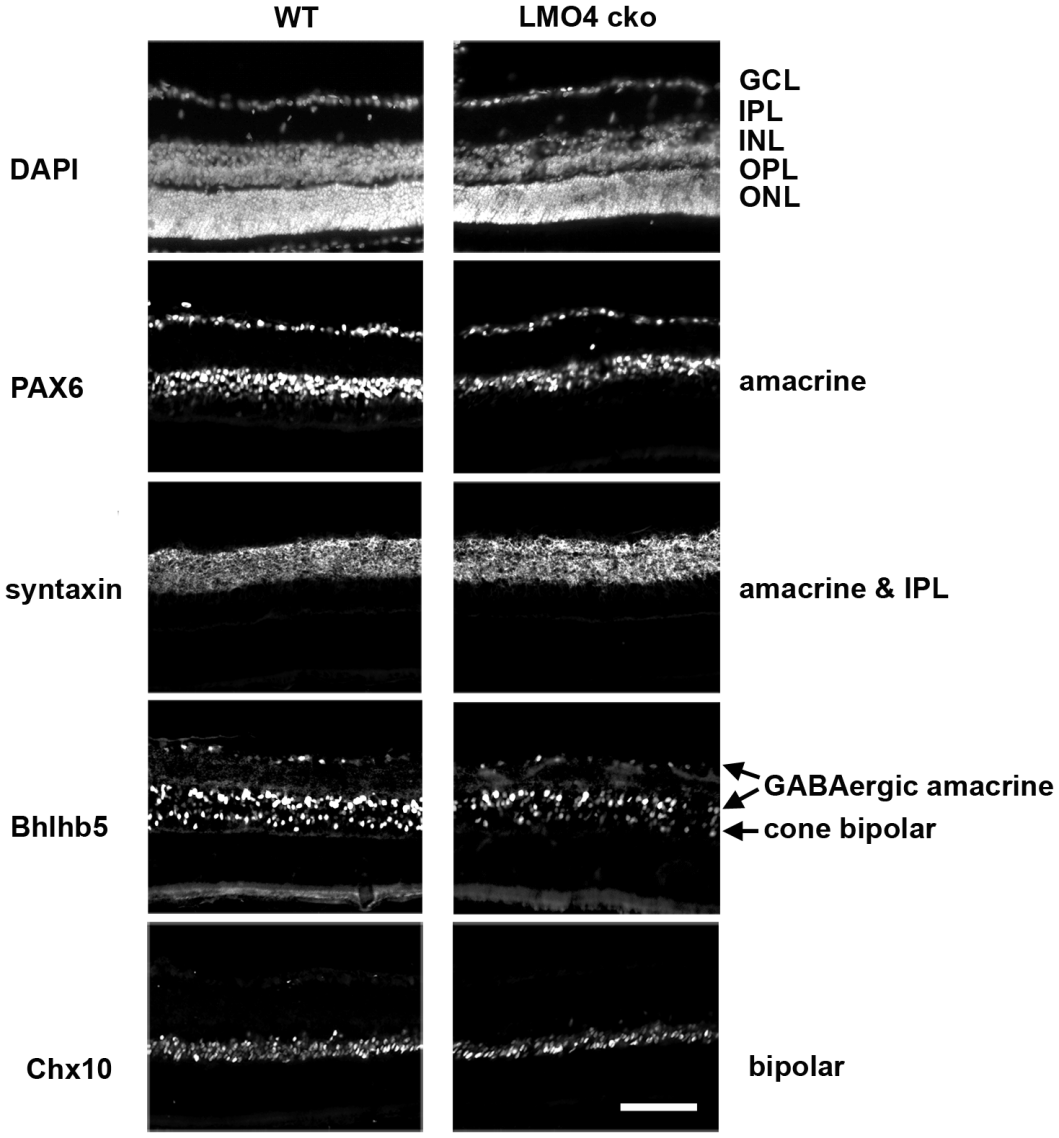
Selective loss of Bhlhb5 amacrine cells and cone bipolar cells in the peripheral retina of LMO4 cko mice. Neuron-specific markers were used to phenotype peripheral retinas at P15 to reveal the functional consequences of LMO4 ablation in retinal progenitors. These are representative sections of those used for quantitation in Figure 6. DAPI stained all retinal nuclei of the ganglionic cell layer (GCL), inner plexiform layer (IPL), inner nuclear layer (INL), outer plexiform layer (OPL), outer nuclear layer (ONL); Pax6 labeled all amacrine cells; Syntaxin labels amacrine cell bodies and the IPL; Bhlhb5 labels GABAergic amacrine cell and OFF-cone bipolar cell nuclei; Chx10 labels bipolar cell nuclei. A significant reduction in Pax6+, Bhlhb5+, Chx10+ cells was observed in LMO4 cko mice. Scale bars, 100 µm.

**Figure 5:**
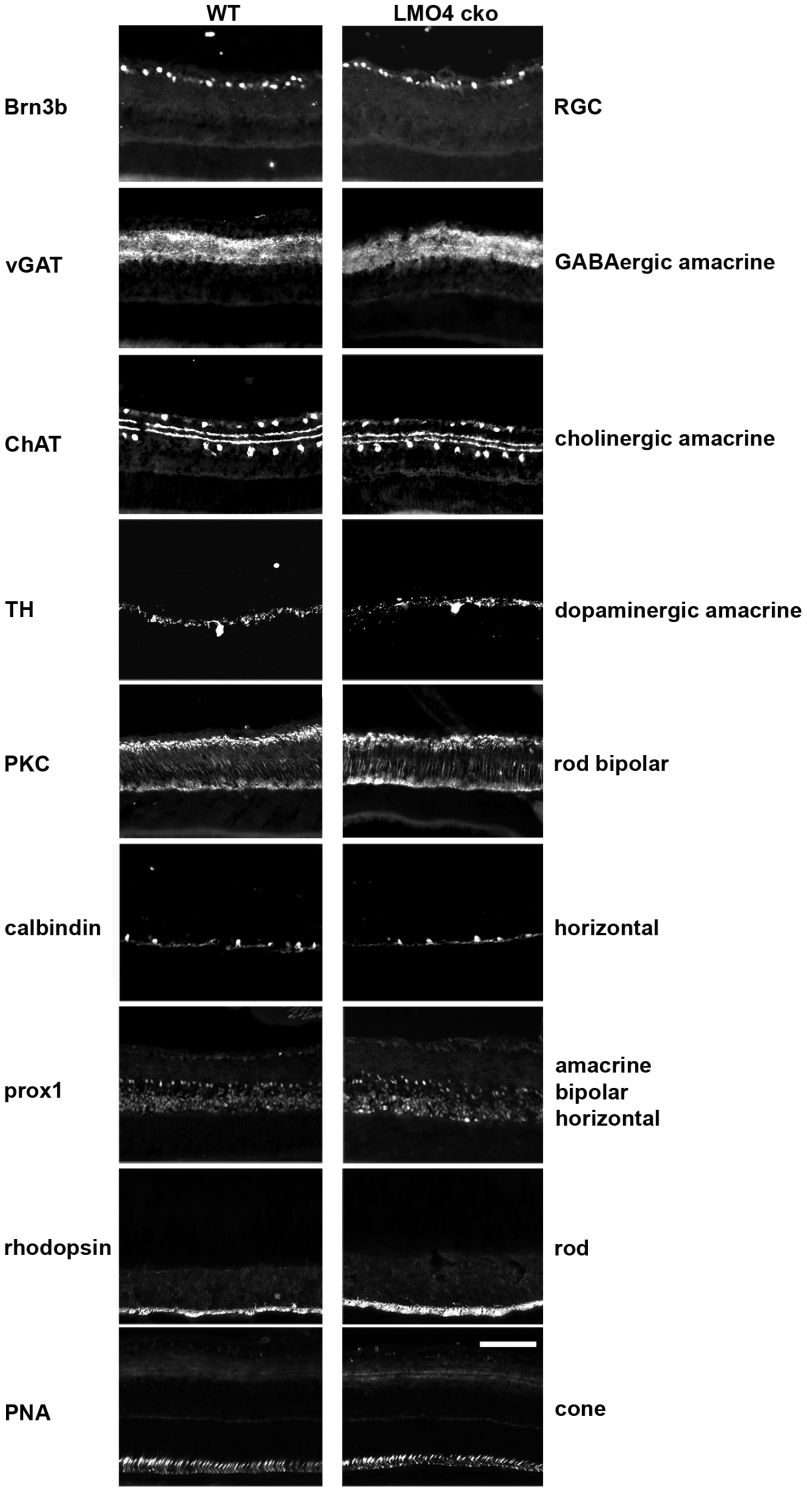
Ganglion cells, cholinergic and dopaminergic amacrine cells, rod bipolar and horizontal cells appear normal in the peripheral retina of LMO4 cko mice. No significant difference was seen with immunostaining markers: Brn3b, retinal ganglion cells (RGC); vesicular GABA transporter (vGAT), GABAergic neurons; choline acetyltransferase (ChAT), cholinergic amacrine cells; tyrosine hydroxylase (TH), dopaminergic amacrine cells; PKC, rod bipolar cells, calbindin, horizontal cells; prox1, amacrine, bipolar and horizontal cells; rhodopsin, rod photoreceptors; peanut agglutinin (PNA), cone photoreceptors. Scale bars, 100 µm.

**Figure 6:**
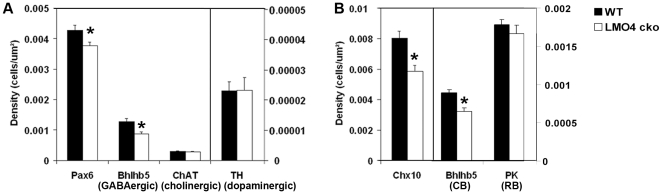
Quantitation of Bhlhb5+ amacrine and bipolar cells in P15 retinas. Immunopositive cells were counted at the peripheral retinas, within 500 µm from the tip, in the plane of the optic nerve. (A) A reduction in the density of amacrine cells labeled with the pan-amacrine Pax6 antibody and Bhlhb5 antibody that labels GABAergic amacrine cells was measured in LMO4 cko mice compared to littermate controls. No difference in cholinergic (ChAT+) or dopaminergic (TH+) amacrine cell density was observed. (B) A reduction in the density of bipolar cells labeled with the pan-bipolar Chx10 antibody and Bhlhb5+ cone bipolar (CB) cells was observed in LMO4 cko mice. However, PKC+ rod bipolar (RB) cell density was not different. Filled bars are for littermate controls and open bars are for LMO4 cko mice. The bars indicate s.e.m. and the asterisks indicate p<0.05.

By immunostaining with cell-type specific markers at P15, we further determined how conditional ablation of LMO4 in retinal progenitors affects development of the “peripheral” retina at the optic nerve plane (Figure 4). Nuclei stained with DAPI showed no obvious anatomical difference in the retina of the LMO4 cko mice compared to littermate controls. The pan-amacrine cell marker Pax6 showed a slight (15%) reduction of amacrine cells in LMO4 cko mice. Using amacrine subtype specific markers, we found a 30% reduction in the number of GABAergic (Bhlhb5^+^) amacrine cells (Figure 4), but no difference in the number of cholinergic (ChAT^+^) or dopaminergic (TH^+^) amacrine cells (Figures 5 & 6A). In addition to amacrine cells localized at the inner region of inner nuclear layer, Bhlhb5 also labels subsets of cone bipolar cells localized at the outer region of the inner nuclear layer [7]. There was also a 30% reduction in the number of Bhlhb5+ cone bipolar cells in LMO4 cko mice compared to littermate controls. In contrast, there was no difference in the number of rod bipolar cells that are immuno-positive for protein kinase C (PKC) (Figures 5 & 6B). Since we observed a 30% reduction in the number of cells labeled with a pan-bipolar cell marker Chx10 (Figures 4 & 6B), this result suggests that in addition to Bhlhb5+ OFF-cone bipolar cells, LMO4 is also required for differentiation of other Bhlhb5-negative cone bipolar cells.

No difference in the number of retinal ganglion cells (Brn3b+), horizontal (calbindin+), rod (rhodopsin+) or cone (peanut agglutinin+, PA+) cells was observed (Figure 5). In addition, the number of photoreceptor nuclei in the outer nuclear layer was not different (data not shown). Taken together, these results indicate a role for LMO4 in the development of Bhlhb5+ (inhibitory GABAergic) amacrine and cone bipolar cells.

**Figure 7:**
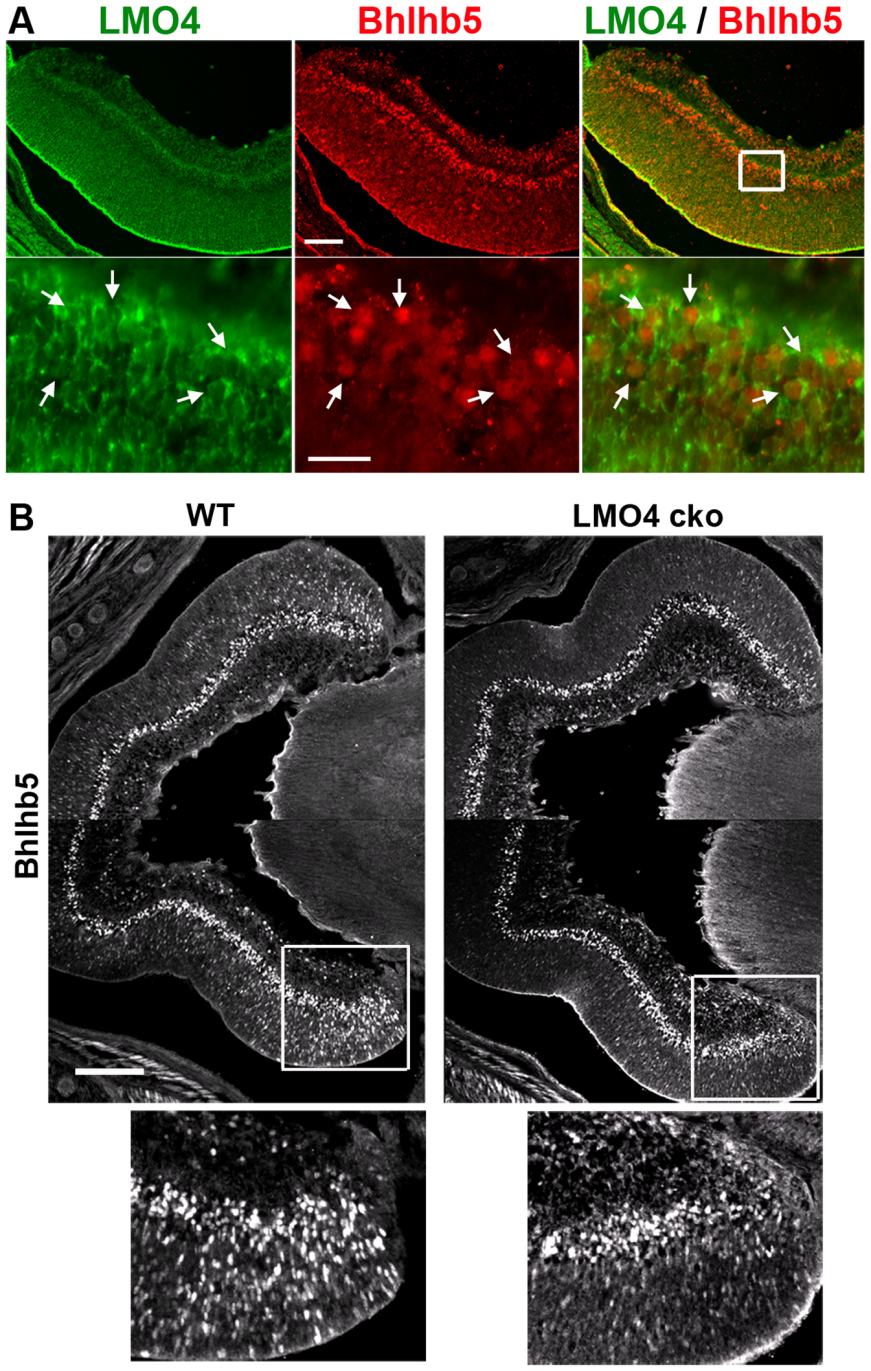
Loss of Bhlhb5+ cells in P0 LMO4 cko retinas. (A) MO4 is colocalized with Bhlhb5 in amacrine cells (arrows). LMO4 mRNA in the cytoplasm was revealed by in situ hybridization with an antisense probe labeled with biotin (green). Bhlhb5 in the nuclei was revealed by an antibody to Bhlhb5 (red). Inset is enlarged in the lower panels. Scale bars = 100 and 25 µm, top and bottom panels, respectively. (B) Bhlhb5 immunostained cells were significantly fewer in the peripheral retina of LMO4 cko mice compared to littermate controls. Inset is enlarged in the lower panels. Scale bar = 100 µm.

Since the LMO4 antibody that worked well in our hands is raised in goat, as is the Bhlhb5 antibody, to compare their expression we performed dual in situ and immunostaining, using biotinylated LMO4 anti-sense mRNA and Bhlhb5 antibody. We found that Bhlhb5 protein was co-expressed with LMO4 mRNA in peripheral developing retinal cells at P0 (Figure 7A). These “peripherally localized” Bhlhb5 amacrine cells were significantly reduced in LMO4 cko mice at P0 (Figure 7B). However, no reduction in Bhlhb5-immunopositive retinal cells was observed at E14.5 in LMO4 cko mice (Figure S2), indicating that loss of LMO4 impairs Bhlhb5^+^ amacrine cell differentiation between E14.5 and P0. To test the possibility that GABAergic amacrine and OFF-cone bipolar subtypes were initially generated but later died of apoptosis in LMO4 cko retinas, retinal sections were immunostained with anti-activated-caspase-3. No increase in apoptotic cells in LMO4 cko retinas was detected at P0 (Figure S3). Taken together, targeted deletion of LMO4 specifically diminished the generation of selective Bhlhb5+ OFF-cone bipolar cells and GABAergic amacrine cells.

To address whether reduced expression of Bhlhb5 was related to loss of LMO4, we cloned a fragment of the mouse Bhlhb5 promoter and tested the effect of LMO4 knock-down on a luciferase reporter by transient transfection of cultured neuronal cells (Figure S4). Bhlhb5 promoter activity was specifically reduced by 50% when LMO4 was knocked down by shRNA, and this mechanism may contribute to reduced numbers of Bhlhb5+ neurons in LMO4 cko retinas.

### Impaired retinal response in α-Cre/LMO4flox mice

**Figure 8:**
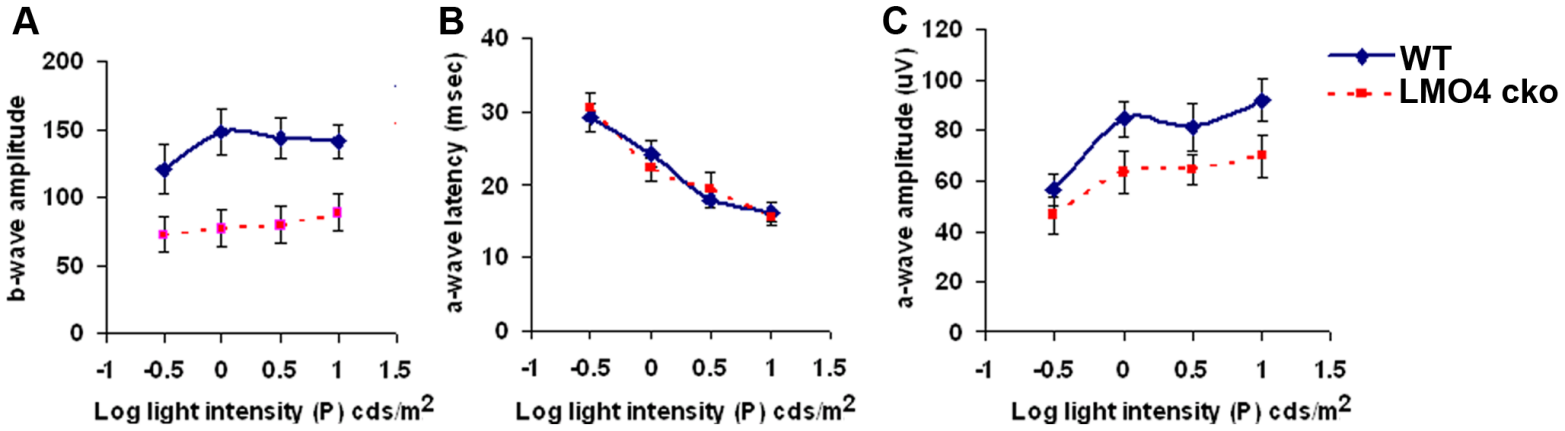
Electroretinography reveals a deficit in the b-wave in LMO4 cko mice. ERGs were recorded from 8 week old littermate control (diamonds and solid line) and LMO4 cko (squares and dashed line) mice. (A) b-wave average amplitude was significantly reduced at all light intensities. (B) a-wave amplitude tended to be lower in LMO4 cko mice, but was not significantly different. (C) a-wave latency was not different between control and LMO4 cko mice. All measures are means ± SEM for n = 13 control (WT) and n = 12 LMO4 cko mice.

To test whether the reduction of Bhlhb5+ GABAergic amacrine cells in LMO4 cko mice affects retinal function, we performed electroretinography (ERG) on dark-adapted adult LMO4 cko or littermate control mice. ERG measures the electrical response of the various components of the retina to light stimulation. The response normally comprises a hyperpolarization “a-wave” and a depolarization “b-wave” that measure photoreceptor and inner layer function, respectively. ERG recording revealed a significant reduction in the amplitude of the b-wave (Figure 8A), consistent with the loss of Bhlhb5 amacrine cells. Both the amplitude and latency of the a-wave (Figure 8B, C) were not affected (Figure 8C) consistent with similar photoreceptor cell numbers in LMO4 cko and littermate controls.

In addition, to compare the function of the cone pathways, we also measured the light-adapted, long-flash (i.e., ON-OFF) ERG. This method uses a background photopic light to saturate the rods, effectively isolating cone photoreceptor pathways; and long duration flashes are used to separate responses from downstream ON- and OFF- bipolar cells with ON-bipolar being favored at flash onset and OFF-bipolar cells at offset. Despite a reduction in Bhlhb5+ type 2 Off-cone bipolar cells [7], we did not observe a difference in ON- or OFF-bipolar cell responses of LMO4 cko mice (data not shown).

## Discussion

Germline ablations of essential transcription regulatory factors that cause embryonic lethality preclude the analysis of their function at later developmental stages. This issue is especially problematic for the study of perinatal differentiation. In particular, the development of the retina is not complete until two weeks after birth. Here, we selectively ablated LMO4 in peripheral retinal progenitors and found that LMO4 is required for visual function and specifically, the development of GABAergic amacrine cells and type 2 OFF-cone bipolar cells.

Our observation of coloboma in LMO4 germline deleted mice, reported here for the first time, lead us to explore the function of LMO4 in retinal development. We found that coloboma was not always linked to exencephaly, suggesting that the impaired mechanisms that underlie these phenotypes might be separable or only partially penetrant. In contrast to the increased eye size observed in zebrafish by morpholino knockdown of the LMO4b gene [27], we often observed smaller eyes in LMO4 null mice. It should be noted that the sequence of the zebrafish LMO4a gene, rather than LMO4b, is more similar to LMO4 of mammals. Whether the different phenotype in zebrafish represents a species-specific difference or the partial redundancy of the two LMO4 duplicated genes of zebrafish is not known.

Amacrine cells in the retina constitute an assortment of different subtypes of interneurons that perform highly complex information processing. The transcription factors that control the differentiation of amacrine cells are gradually being revealed from genetic models. Here, we showed that LMO4 is co-expressed with Pax6, a pan amacrine cell marker, and by selective ablation of LMO4 in retinal progenitor cells we demonstrated that LMO4 is required for Bhlhb5+ amacrine cell development.

A 30% reduction in the number of Bhlhb5+ amacrine cells was observed in the peripheral retina of LMO4 cko mice. This suggests the existence of two subsets of Bhlhb5+ neurons, one that is LMO4-dependent and another that is LMO4-independent. In addition, the deficit appears to affect the postnatal wave of Bhlhb5+ neurons suggesting a temporal requirement for lmo4 in retinal neuron development. Despite the loss of Bhlhb5+ (GABAergic) amacrine cells, immunostaining for glutamic acid decarboxylase (GAD), an enzyme that catalyzes the decarboxylation of glutamate to GABA neurotransmitter, did not reveal any difference between LMO4 cko and littermate control mice. This might reflect a compensatory effect of existing Bhlhb5 neurons. In either case, the 30% reduction of Bhlhb5+ amacrine cells in LMO4 cko mice was sufficient to impact the retinal visual signals as shown by ERG. The reduced b-wave amplitude is consistent with loss of amacrine cells (and cone bipolar cells). Although, given that Bhlhb5+ amacrine cells are typically GABAergic inhibitory interneurons, the reduction, rather than the elevation of b-wave amplitude, was unexpected. However, it is possible that the function of these Bhlhb5+ GABAergic interneurons is to inhibit other inhibitory interneurons.

This is the first report that links LMO4 with GABAergic amacrine cell development and function. A previous study reported a role for LMO4 in regulating the balance of inhibitory and excitatory interneurons that develop in the spinal cord; ablation of LMO4 lead to a loss of V2b GABAergic inhibitory interneurons and favored V2a glutamatergic excitatory interneurons [20]. However, unlike in the spinal cord, we did not observe an increase in the excitatory cholinergic amacrine cells despite the loss of GABAergic inhibitory amacrine cells. Thus, LMO4 appears to have different functions in different regions of the nervous system.

Similar to its requirement for inhibitory amacrine interneuron development in the eye, Bhlhb5 is also required for the survival of a subtype of inhibitory interneurons in the spinal cord that regulates pruritis; loss of inhibitory synaptic input in the Bhlhb5 null mice results in an excessive abnormal itch [35]. In contrast, in the cerebral cortex Bhlhb5 specifically regulates the differentiation of excitatory glutamatergic projection neurons of neocortical layers II–V and is required for structural organization in the somatosensory and caudal motor cortices [36]. LMO4 is also expressed in the layer II/III and V cortical neurons [37], [38] and is critical for patterning thalamocortical connections during development [13]. In light of our finding that LMO4 is required for Bhlhb5+ retinal neuron development, the similar spatial and temporal expression patterns of LMO4 and Bhlhb5 make it plausible that LMO4 regulates Bhlhb5 expression in the developing cortex and participates in the development of glutamatergic projection neurons.

Our study found no deficit in photoreceptor cell number and function as reflected by a normal a-wave amplitude and latency. Consistent with our finding, the Bhlhb5-null mutation does not affect photoreceptor cell development [39].

Another important finding of our study is that LMO4 contributes to the differentiation of Bhlhb5+ type 2 OFF-cone bipolar cells, but not the rod bipolar cells whose development, on the other hand, depends on bHLHb4 [10]. Despite a 30% reduction of Bhlhb5+ cone bipolar cells observed in LMO4 cko mice, the response of the ON-OFF ERG was not different between LMO4 cko and littermate controls. Although this result would argue against a role for LMO4 in cone bipolar cell function, alternatively, there is loss in both ON- and Off- bipolar cells, as reflected in 30% reduction in the number of Chx10+ bipolar cells.

The basic helix-loop-helix transcription factors Math3+ and NeuroD+ are important for amacrine cell development, whereas the Math3+ and Mash1+ are for the type 2 OFF-cone bipolar cells [39]. Previous studies showed that LMO4 forms a complex with the basic helix-loop-helix transcription factor SCL and GATA2 and to regulate interneuron differentiation in the spinal cord [20]. Our studies show that the Bhlhb5 promoter is dependent upon LMO4 for expression in neuronal cells in culture. However, since LMO4 is not known to bind DNA directly, the effects of LMO4 on Bhlhb5 promoter activity are likely to be indirect. Future studies will be required to determine whether LMO4 interacts with these bHLH transcription factors to activate Bhlhb5 expression in the retina.

## Supporting Information

Figure S1 Expression of the green fluorescent protein (GFP) reporter from the Pax6 α-enhancer in αCre-IRES-EGFP mice. At embryonic day 14.5 (E14.5), GFP is highly expressed in the peripheral retina, predominantly in the ventral compartment. By postnatal day 0 (P0), GFP expression is widespread in the amacrine cell layer. GFP expression is an indicator of where Cre-recombinase is expressed.
(0.99 MB TIF)

Figure S2 Bhlhb5 expression at E14.5 is not different in LMO4 cko mice. Immunofluorescent labeling of Bhlhb5 retinal neurons is compared from wild type (WT) and LMO4 cko retinas at E14.5. Bhlhb5 in red; DAPI, and mitotic marker phosphorylated histone 3 (PH3), in blue as indicated; GFP reporter expression driven by the Pax6 α-enhancer, in green. Most of the Bhlhb5 expression was detected in non-mitotic cells. No significant difference was observed in Bhlhb5+ differentiated cells or PH3+ mitotic cells in LMO4 cko retinas. Scale bar, 100 µm.
(3.12 MB TIF)

Figure S3 No evidence for increased cell death at P0 in LMO4 cko retinas. Antibody to activated caspase 3 revealed a similar number of apoptotic neurons in the retinas of littermate control (WT) and LMO4 cko mice (Arrows). Scale bar, 100 µm.
(0.41 MB TIF)

Figure S4 Knockdown of LMO4 reduced Bhlhb5 promoter activity. (A) Western blot immunostained for anti-Flag antibody shows the efficacy of LMO4shRNA to knockdown LMO4 expression in transiently transected F11 cells expressing exogenous Flag-tagged LMO4. (B). LMO4-specific silencing shRNA (LMO4shRNA) reduced the Bhlhb5 promoter-dependent luciferase activity in F11 neuronal cells. In contrast, the non-silencing control shRNA (CtlshRNA) had no effect. Empty vector only (vector) was also used as a control for shRNA. Mean luciferase activities, normalized to a cotransfected beta-gal reporter, are shown with standard error of mean (n = 3 independent experiments, each with 3 replications. *, p<0.05).
(0.63 MB TIF)
