# Supplementary figures and images for: Loss of LMO4 in the Retina Leads to Reduction of GABAergic Amacrine Cells and Functional Deficits
Source: PLoS One. 2010 Oct 7;5(10):e13232. doi: 10.1371/journal.pone.0013232 (PMC2951357; doi:10.1371/journal.pone.0013232)

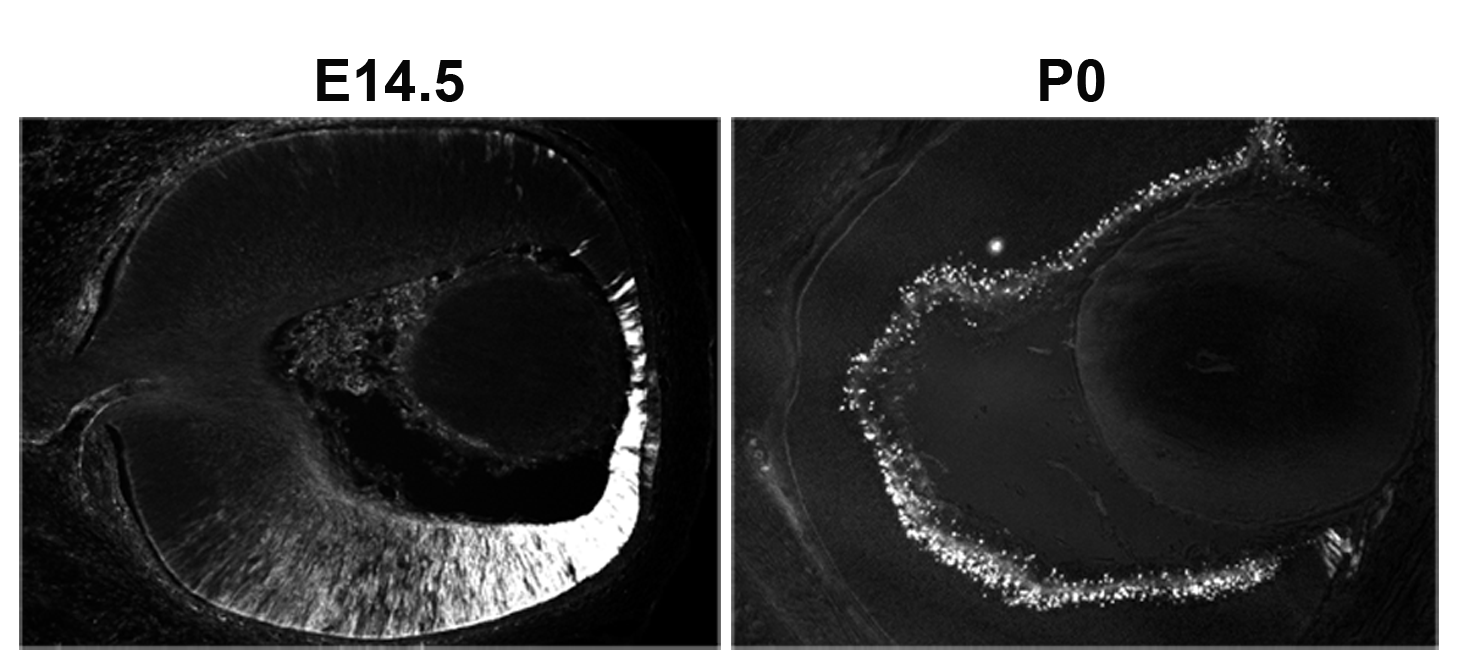

Supplement: Figure S1 — Expression of the green fluorescent protein (GFP) reporter from the Pax6 α-enhancer in αCre-IRES-EGFP mice. At embryonic day 14.5 (E14.5), GFP is highly expressed in the peripheral retina, predominantly in the ventral compartment. By postnatal day 0 (P0), GFP expression is widespread in the amacrine cell layer. GFP expression is an indicator of where Cre-recombinase is expressed. (0.99 MB TIF) [file pone.0013232.s001.tif]

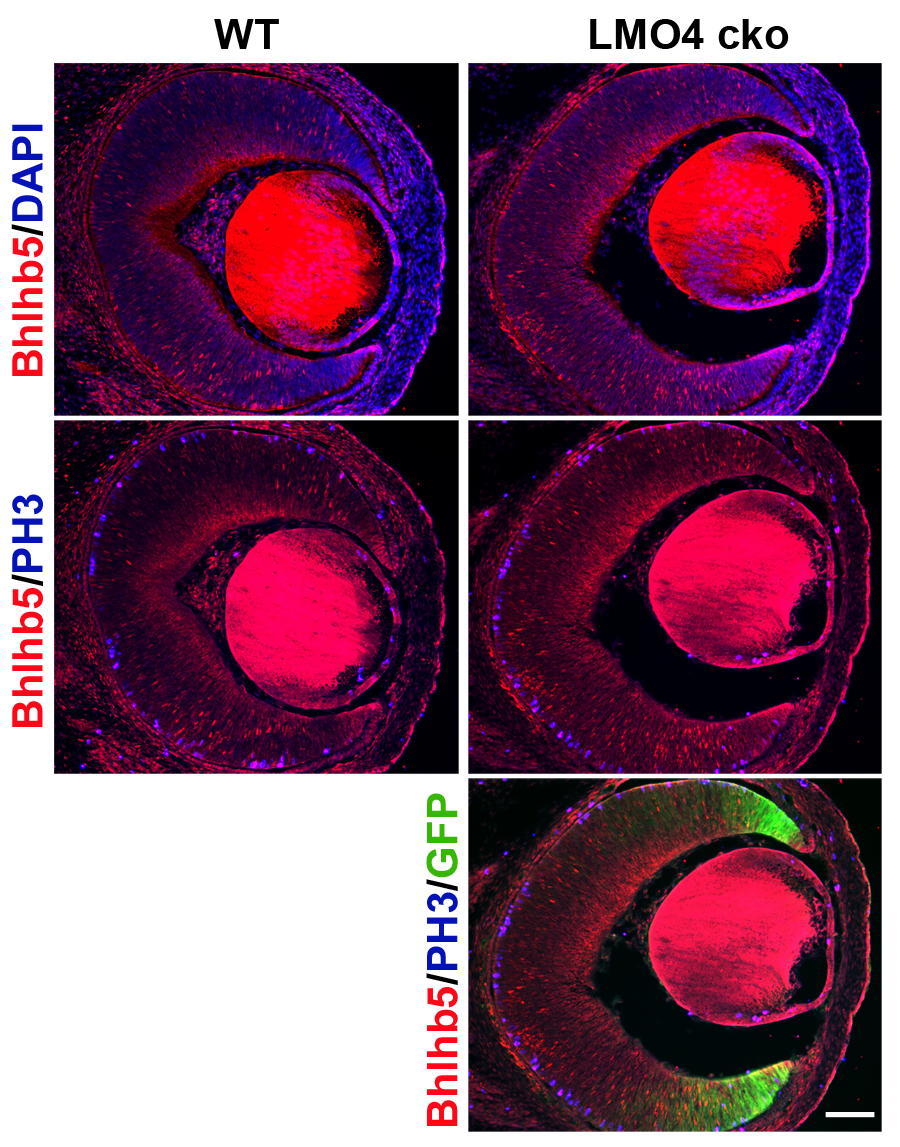

Supplement: Figure S2 — Bhlhb5 expression at E14.5 is not different in LMO4 cko mice. Immunofluorescent labeling of Bhlhb5 retinal neurons is compared from wild type (WT) and LMO4 cko retinas at E14.5. Bhlhb5 in red; DAPI, and mitotic marker phosphorylated histone 3 (PH3), in blue as indicated; GFP reporter expression driven by the Pax6 α-enhancer, in green. Most of the Bhlhb5 expression was detected in non-mitotic cells. No significant difference was observed in Bhlhb5+ differentiated cells or PH3+ mitotic cells in LMO4 cko retinas. Scale bar, 100 µm. (3.12 MB TIF) [file pone.0013232.s002.tif]

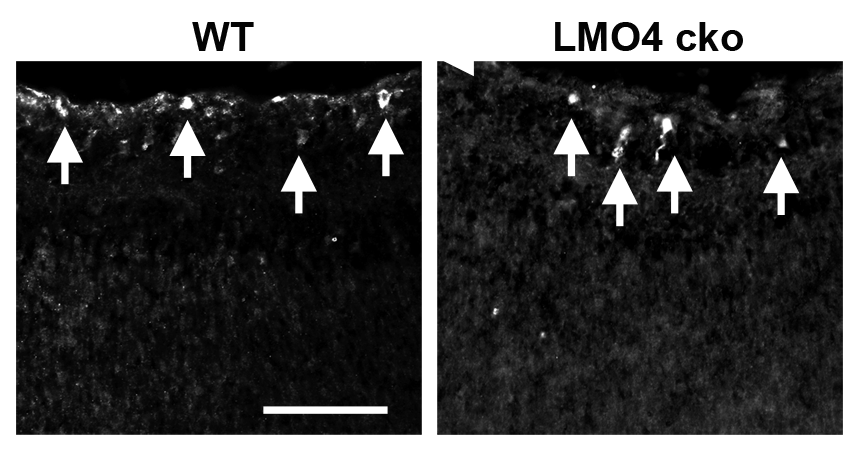

Supplement: Figure S3 — No evidence for increased cell death at P0 in LMO4 cko retinas. Antibody to activated caspase 3 revealed a similar number of apoptotic neurons in the retinas of littermate control (WT) and LMO4 cko mice (Arrows). Scale bar, 100 µm. (0.41 MB TIF) [file pone.0013232.s003.tif]

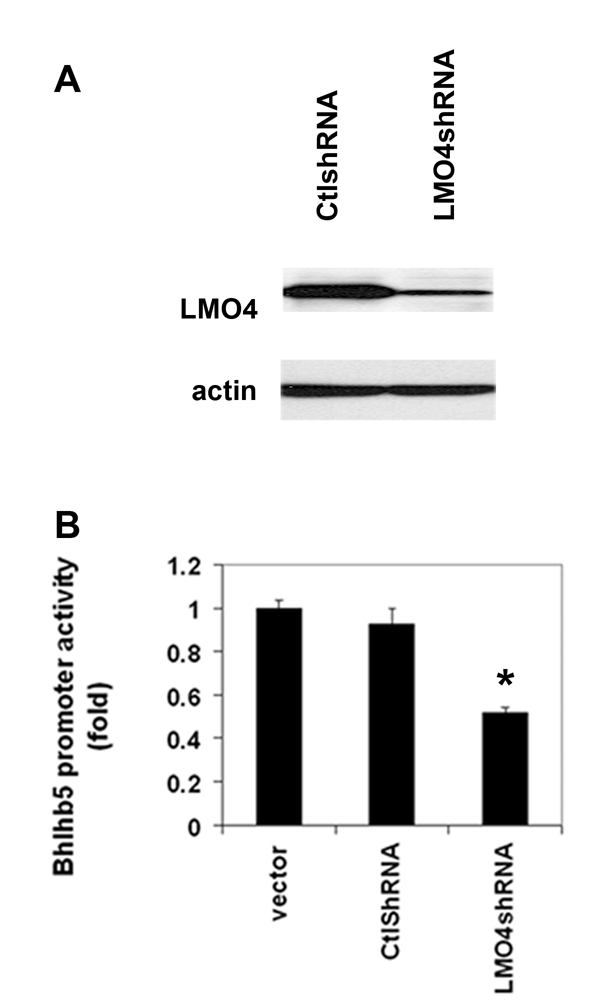

Supplement: Figure S4 — Knockdown of LMO4 reduced Bhlhb5 promoter activity. (A) Western blot immunostained for anti-Flag antibody shows the efficacy of LMO4shRNA to knockdown LMO4 expression in transiently transected F11 cells expressing exogenous Flag-tagged LMO4. (B). LMO4-specific silencing shRNA (LMO4shRNA) reduced the Bhlhb5 promoter-dependent luciferase activity in F11 neuronal cells. In contrast, the non-silencing control shRNA (CtlshRNA) had no effect. Empty vector only (vector) was also used as a control for shRNA. Mean luciferase activities, normalized to a cotransfected beta-gal reporter, are shown with standard error of mean (n = 3 independent experiments, each with 3 replications. *, p<0.05). (0.63 MB TIF) [file pone.0013232.s004.tif]
